# Small cats in big trouble? Diet, activity, and habitat use of jungle cats and leopard cats in threatened dry deciduous forests, Cambodia

**DOI:** 10.1002/ece3.7316

**Published:** 2021-03-30

**Authors:** Susana Rostro‐García, Jan F. Kamler, Christin Minge, Anthony Caragiulo, Rachel Crouthers, Milou Groenenberg, Thomas N. E. Gray, Visattha In, Chanratana Pin, Prum Sovanna, Marc Kéry, David W. Macdonald

**Affiliations:** ^1^ Department of Zoology Wildlife Conservation Research Unit University of Oxford The Recanati‐Kaplan Centre Abingdon United Kingdom; ^2^ Institute of Ecology and Evolution Friedrich‐Schiller University of Jena Germany; ^3^ Sackler Institute for Comparative Genomics American Museum of Natural History New York NY USA; ^4^ World Wild Fund for Nature Cambodia Phnom Penh Cambodia; ^5^ Tigers Alive Initiative WWF‐Malaysia Kuala Lumpur Malaysia; ^6^ Ministry of Environment Phnom Penh Cambodia; ^7^ Swiss Ornithological Institute Sempach Switzerland

**Keywords:** activity overlap, biomass consumed, camera‐trapping, dietary overlap, *Felis chaus*, open dry deciduous forests, *Prionailurus bengalensis*, Southeast Asia, species interactions, two‐species occupancy modeling

## Abstract

Dry deciduous dipterocarp forests (DDF) cover about 15%–20% of Southeast Asia and are the most threatened forest type in the region. The jungle cat (*Felis chaus*) is a DDF specialist that occurs only in small isolated populations in Southeast Asia. Despite being one of the rarest felids in the region, almost nothing is known about its ecology. We investigated the ecology of jungle cats and their resource partitioning with the more common leopard cats (*Prionailurus bengalensis*) in a DDF‐dominated landscape in Srepok Wildlife Sanctuary, Cambodia. We used camera‐trap data collected from 2009 to 2019 and DNA‐confirmed scats to determine the temporal, dietary and spatial overlap between jungle cats and leopard cats. The diet of jungle cats was relatively diverse and consisted of murids (56% biomass consumed), sciurids (15%), hares (*Lepus peguensis*; 12%), birds (8%), and reptiles (8%), whereas leopard cats had a narrower niche breadth and a diet dominated by smaller prey, primarily murids (73%). Nonetheless, dietary overlap was high because both felid species consumed predominantly small rodents. Both species were primarily nocturnal and had high temporal overlap. Two‐species occupancy modelling suggested jungle cats were restricted to DDF and had low occupancy, whereas leopard cats had higher occupancy and were habitat generalists. Our study confirmed that jungle cats are DDF specialists that likely persist in low numbers due to the harsh conditions of the dry season in this habitat, including annual fires and substantial decreases in small vertebrate prey. The lower occupancy and more diverse diet of jungle cats, together with the broader habitat use of leopard cats, likely facilitated the coexistence of these species. The low occupancy of jungle cats in DDF suggests that protection of large areas of DDF will be required for the long‐term conservation of this rare felid in Southeast Asia.

## Graphical abstract

We studied the ecology of jungle cat, one of the rarest felids in Southeast Asia, and its resource partitioning with the more common leopard cat. The diet, activity, and habitat use overlapped considerably between the two felid species, suggesting high‐niche overlap. Overall, we found that jungle cats were habitat specialists for open dry deciduous forests and were rare, suggesting that large areas of this habitat will be required for their long‐term conservation.

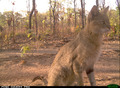

## 1 INTRODUCTION

Southeast Asia is considered both one of the most important biodiversity hotspots and one of the most biologically threatened regions worldwide (Hughes, 2017). This region, which is amidst a conservation crisis, supports more threatened species than any other continental area, including the highest proportion of mammals categorized as threatened (Duckworth et al., 2012; Gray et al., 2018). Thus, there is an urgent need of multifaceted information about the species inhabiting Southeast Asia, including knowledge about their ecology, threats to their survival, and interactions with other species. Nonetheless, Southeast Asian wildlife has been considerably understudied, and the lack of information together with rapid drivers of species loss that characterize this region, make protecting biodiversity highly challenging (Hughes, 2017). To date, most studies and conservation efforts in Southeast Asian mammals have focused primarily on large flagship and high‐profile species, such as tigers (*Panthera tigris*; Walston et al., 2010), orangutans (*Pongo* spp., Pandong et al., 2019), Asian elephants (*Elephas maximus*; Wadey et al., 2018) and more recently leopards (*P. pardus*; Rostro‐García, Kamler, et al., 2016), and clouded leopards (*Neofelis nebulosa*; Macdonald et al., 2019), with little attention given to smaller species (Brodie, 2009).

Southeast Asia is the most felid‐rich region in the world, supporting 12 of the 37 recognized extant species (Luo et al., 2014). Eight of nine felid species inhabiting the Southeast Asian mainland occur in the closed canopy evergreen and semi‐evergreen forests (hereafter evergreen forests) that historically dominated the region (Duckworth et al., 2014; Francis, 2019). Consequently, nearly all previous research on felid communities has been conducted in evergreen forests (Duckworth et al., 2014). However, dry deciduous dipterocarp forests (DDF), characterized by an open canopy and a grassy understory, support high mammalian biomass and are globally irreplaceable for numerous species, including several classified as threatened (Tordoff et al., 2005; Wohlfart et al., 2014). The DDF, which currently covers about 15%–20% of Southeast Asia, are now the most threatened of all forest types of the region (Pin et al., 2020; Wohlfart et al., 2014). In contrast to evergreen forests, there has been little research on felid species in DDF.

In Southeast Asia, four species of felids are known to occupy DDF: the tiger, which is now extirpated in the eastern half of its distribution (O’Kelly et al., 2012; Rasphone et al., 2019), the Indochinese leopard (*P. pardus delacouri*), which is classified as Critically Endangered (Rostro‐García et al., ,^2018^, ^2019^), the jungle cat (*Felis chaus*) and the leopard cat (*Prionailurus bengalensis*). The jungle cat appears to be the only felid that is a DDF specialist in Southeast Asia (Duckworth et al., 2005) because tigers, leopards, and leopard cats are habitat generalists that also live in evergreen forests (Duckworth et al., 2014). Although jungle cats are relatively common in India and southwestern Asia, this species has likely suffered drastic declines in Southeast Asia due to habitat modification within DDF landscapes and the pervasive indiscriminate snaring in the region (Gray et al., 2016). Therefore, the jungle cat now occurs only in small and isolated populations, making it one of the rarest felids in mainland Southeast Asia (Duckworth et al., 2005), along with the flat‐headed cat (*Prionailurus planiceps*) and fishing cat (*Prionailurus viverrinus*). This rarity appears to be a relatively recent phenomenon because the jungle cat was described as common in the region by Lekagul and McNeely (1977), but it is now seldom encountered (Gray et al., 2016). Almost nothing is known about the ecology of jungle cats in Southeast Asia, with the majority of studies on the species being conducted in India and southwestern Asia (Gray et al., 2016; Table 1). In addition, resource partitioning between jungle cats and other felids has never been studied in the region, so the mechanisms that facilitate their coexistence with other felids are unknown.

**TABLE 1:** Summary of dietary studies of jungle cats (*Felis chaus*), with sample sizes > 10 scats. The DNA column indicates if genetic analysis was used on scats to confirm species

Country – Site	Sample size	DNA	Top prey categories^a^
Cambodia			
Srepok Wildlife Sanctuary^b^	17	Yes	Murid (56%), sciurid (15%), hare (12%), bird (8%), reptile (8%)
India			
Bandipur Tiger Reserve^c^	67	No	Murid (64%), bird (13%), hare (10%), lizard (7%), chital fawn (4%)
Kanha Tiger Reserve^d^	27	No	Murid (100%), lizard (7%)
Pench Tiger Reserve^e^	85	No	Rodent (64%), hare (11%), reptile (8%), bird (7%), chital (6%)
Sariska Tiger Reserve^f^	287	No	Rodent (39%), hare (29%), bird (17%), cattle (16%), chital (7%)
Sariska Tiger Reserve^g^	69	No	Rodent (74%), bird (42%), reptile (26%), insect (23%), wild ungulate (12%)
Pakistan			
Farmland in Punjab^h^	30	No	Rodent (70%), bird (10%), herpetofauna (10%)
Tajikistan			
Near Amu Darya River^i^	100	No	Bird (36%), rodent (34%), hare (13%), fruit (6%)
Uzbekistan			
Aral‐Paygambar Island^j^	379	No	Murid (63%), hare (22%)
Aral‐Paygambar Island^k^	472	No	Murid (89%), bird (28%), insect (23%), hare (14%), fruit (9%), reptile (8%)
Lower Amu Darya River^l^	33	No	Rodents (63%), birds (31%)

^a^ If seasonal results were given, then an average of the seasonal results was used.

^b^ This study.

^c^ Johnsingh (1983).

^d^ Schaller (1967).

^e^ Majumder et al. (2011).

^f^ Gupta (2011).

^g^ Mukherjee et al. (2004).

^h^ Khan and Beg (1986).

^i^ Chernyshev (1958), as cited in Heptner and Sludskii (1992) (includes contents from 33 stomachs).

^j^ Volozheninov (1972) (includes contents from 15 stomachs).

^k^ Ishunin (1965), as cited in Heptner and Sludskii (1992).

^l^ Allayarov (1964) (includes contents from 6 stomachs).

Competition among carnivores is driven by body size, predatory behavior, diet, and taxonomic similarity, although similarity in body size is the most important factor (Donadio & Buskirk, 2006). Consequently, competition between sympatric carnivores is expected to be most intense between species that are more similar in body size, with the larger species dominating the smaller species. In turn, smaller carnivores employ different avoidance mechanisms to coexist with the next largest carnivore species, including dietary, temporal, and spatial partitioning (Fedriani et al., 1999; Kamler et al., 2003, 2012), although the degree of partitioning may depend on the availability of food resources (Holt & Polis, 1997; Kamler et al., 2007). In particular, dietary overlap often drives interference competition within carnivore guilds, therefore food niche differences often are necessary for successful coexistence (Tsunoda et al., 2017). Previous studies have shown that smaller felids partition food resources with larger felids by consuming smaller prey (Harihar et al., 2011; Karanth & Sunquist, 1995; Moreno et al., 2006; Nagy‐Reis et al., 2019), thereby demonstrating that food partitioning is used to help facilitate coexistence between species. Another mechanism that may facilitate coexistence of ecologically similar species is spatial partitioning, particularly when food resources are limited and dietary overlap is high. In such cases, subordinate species may use different habitat types to avoid potential encounters with dominant species and thereby reduce interference competition (Foster et al., 2013; Horne et al., 2009; Scognamillo et al., 2003). Alternatively, when overlap levels of spatial niche and food resources are high, temporal partitioning can occur, whereby subordinate species adjust their activity patterns to reduce encounters and facilitate coexistence with a dominant competitor. Low overlap of activity patterns has been shown to exist between small felid species in Southeast Asia (Kamler, Inthapanya, et al., 2020; Lynam et al., 2013; McCarthy et al., 2015; Mukherjee et al., 2019; Rasphone et al., 2020) and in other regions of the world (Leonard et al., 2020; Lucherini et al., 2009; Nagy‐Reis et al., 2019), indicating this strategy also is used to enable coexistence between similarly sized felids.

In Southeast Asia, the coexistence mechanisms employed by sympatric felids in DDF might differ from those used in evergreen forests due to the more open habitat. For example, spatial and temporal avoidance might be stronger in more open habitats, where reduced herbaceous cover might otherwise increase encounter rates between felid species. Furthermore, exploitive or interference competition between small felids might be particularly strong in DDF, which exhibits a harsh dry season during which the grassy understory annually burns, most water sources dry up, and small vertebrate prey decrease (Kamler et al., 2021; McShea & Davies, 2011; Pin et al., 2020; Walker & Rabinowitz, 1992), thereby causing a potential seasonal spike in food competition. The jungle cat (4–6 kg; Francis, 2019) reportedly preys mostly on species < 1 kg (Sunquist & Sunquist, 2002), such as murids and other small rodents, as well as on hares (*Lepus* spp.), birds, and small reptiles (Table 1). In India, the jungle cat reportedly also regularly consumes livestock and wild ungulates (Table 1), presumably as carrion (Gupta, 2011), but also via predation on fawns (Nowell & Jackson, 1996; Sunquist & Sunquist, 2002). However, the diet of the jungle cat in Southeast Asia is unknown. Anecdotal reports and limited data suggest the jungle cat is more diurnal than other felids (Gray et al., 2014; Lekagul & McNeely, 1977; Nowell & Jackson, 1996; Sunquist & Sunquist, 2002), although there is a paucity of published data to confirm this.

Leopard cats (2–3 kg; Grassman et al., 2005), which are still common and widespread throughout Southeast Asia (Ross et al., 2015), feed primarily on small rodents weighing < 0.5 kg (Kamler, Inthapanya, et al., 2020). Therefore, dietary overlap and potential competition with jungle cats could be high, unless jungle cats regularly consume larger food items such as carrion or ungulate fawns in DDF, similar to that reported in India (Table 1). Leopard cats reportedly are almost strictly nocturnal (Grassman et al., 2005; Kamler, Inthapanya, et al., 2020; Lynam et al., 2013; Mukherjee et al., 2019; Rasphone et al., 2020), which could facilitate temporal partitioning between these species if jungle cats are more diurnal. Jungle cats are considerably taller than leopard cats (Francis, 2019) and twice the body weight; consequently, jungle cats should behaviorally dominate leopard cats, although their interactions have not been studied.

In this study, we investigated the ecology of jungle cats and their resource partitioning with leopard cats in eastern Cambodia. This area is identified as the last stronghold for jungle cats in Southeast Asia (Gray et al., 2016) and contains one of the largest tracts of threatened DDF remaining in the region (Wohlfart et al., 2014). We used camera‐trap surveys and DNA‐confirmed scats to study the temporal, spatial, and dietary overlap between jungle cats and leopard cats. Based on previous studies, we made the following predictions: (a) dietary overlap between species will be relatively low, owing to consumption of larger prey, including carrion and ungulate fawns, by jungle cats; (b) activity overlap between species will be low, because jungle cats will be diurnal whereas leopard cats will be nocturnal; (c) habitat use will differ between species, because jungle cats will use mostly DDF, whereas leopard cats will use mostly evergreen forests; (d) occupancy will be higher for jungle cats than for leopard cats because DDF dominates our study site, and; (e) leopard cats will spatially avoid jungle cats owing to the larger body size and presumed behavioral dominance of the jungle cat.

## 2 METHODS

### 2.1 Study area

**FIGURE 1:**
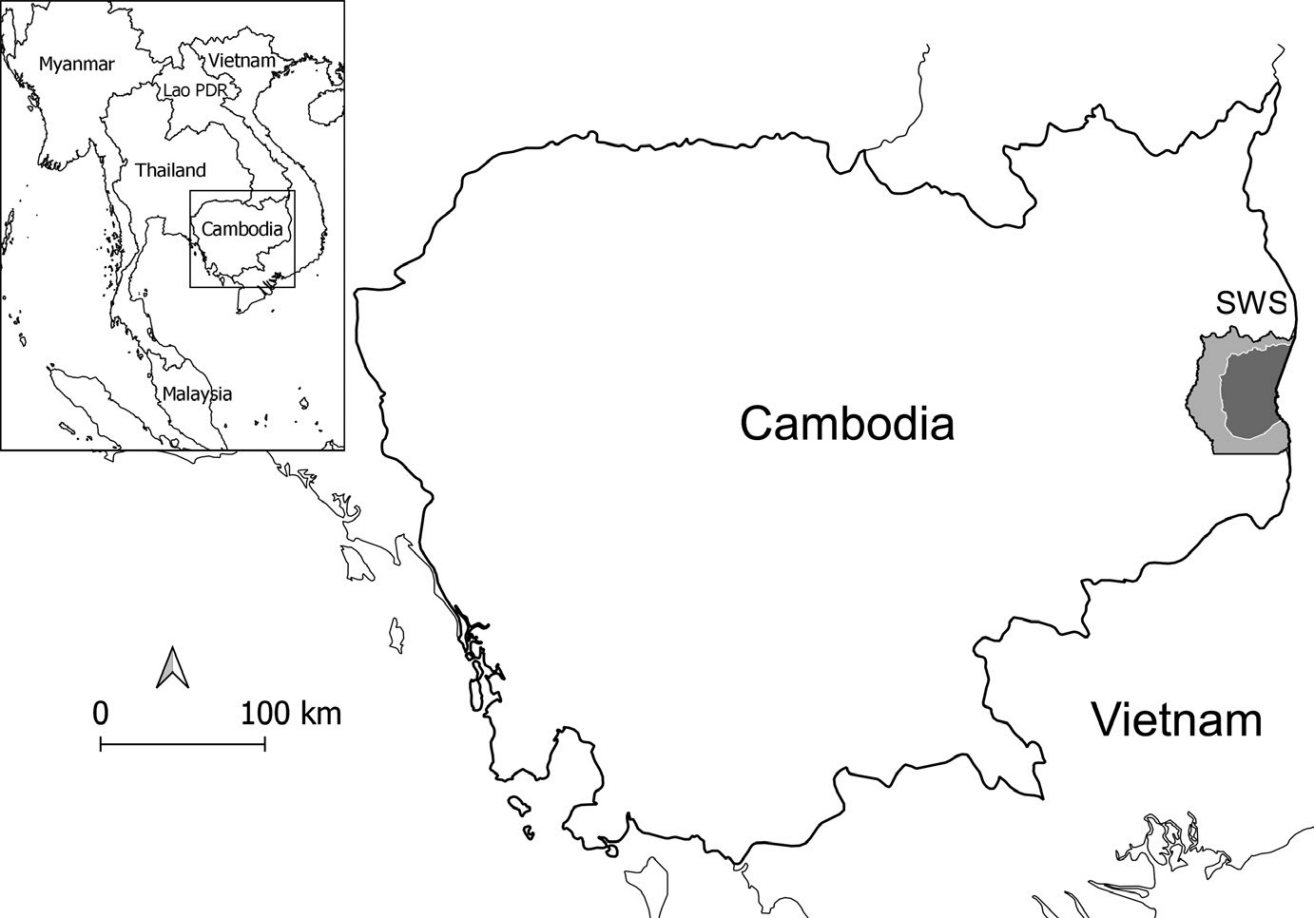
Location of the Srepok Wildlife Sanctuary (SWS) in eastern Cambodia. The dark gray represents the core zone in the eastern part of SWS where this study was conducted

We conducted research in Srepok Wildlife Sanctuary (SWS; 3,730 km^2^), formerly called Mondulkiri Protected Forest until 2016, located in the Eastern Plains Landscape of Cambodia (Figure 1), which comprises one of the three remaining hotspots of DDF in Southeast Asia (Wohlfart et al., 2014). The habitat of SWS is dominated (ca. 70%) by DDF in relatively flat terrain, interspersed with small patches of evergreen forests on hill tops and riparian forests along streams and rivers (Rostro‐García et al., 2018). The DDF in SWS is dominated by two species of Dipterocarpaceae trees, *Shorea obtusa* and *Dipterocarpus tuberculatus*, and an understory of grasses and herbaceous bamboo (*Vietnamosasa* spp.; Pin et al., 2013). The SWS has a distinct dry season from about November to April (average monthly rainfall is 3–121 mm), and a pronounced rainy season from May to October (248–370 mm per month; rainfall data were from nearby Sen Monorom, Cambodia, 1982–2012; climate‐data.org; accessed on 10 July 2019). The DDF is well adapted to the dry season fires (both natural and human‐caused) that occur annually after the dipterocarp trees lose their leaves, which burn most of the grassy understory (McShea & Davies, 2011). The elevation ranges from 100 to 400 m. Large carnivores (> 15 kg) present in SWS during the study period included the leopard, dhole (*Cuon alpinus*), and sun bear (*Helarctos malayanus*; Rostro‐García et al., 2018), although numbers of these species were low. Other smaller carnivores present during the study included the golden jackal (*Canis aureus*), yellow‐throated marten (*Martes flavigula*), small Asian mongoose (*Herpestes javanicus*), crab‐eating mongoose (*H. urva*), ferret‐badger (*Melogale* spp.), large Indian civet (*Viverra zibetha*), small Indian civet (*Viverricula indica*), large‐spotted civet (*Viverra megaspila*), and Asian palm civet (*Paradoxurus hermaphroditus*). The wild ungulate community in SWS is dominated by banteng (*Bos javanicus*), wild pig (*Sus scrofa*), and northern red muntjac (*Muntiacus vaginalis*; Rostro‐García et al., 2018). Our research was carried out within the core zone (ca. 1,700 km^2^), located in the eastern part of SWS, where human access was restricted (Figure 1).

### 2.2 Dietary analysis

The diets of jungle cats and leopard cats were studied by analysis of scats (i.e., feces) collected during the dry seasons from 2013 to 2016. Scats were collected along 30 transects (2 km each) that were established on dirt tracks and trails within the core zone of SWS, as well as opportunistically when conducting other research. For each scat, the scat diameter (when possible), date, and GPS location were recorded. We compared the mean diameter of DNA‐confirmed scats between species using an independent samples *t* test. We obtained approximately 5 g of flakes from the outer coating of the scats and sent them to the Sackler Institute for Comparative Genetics, American Museum of Natural History (New York) for species identification based on mitochondrial DNA analysis (see Caragiulo et al., 2014 for methodological details) using the leopard cat and jungle cat primers from Mukherjee et al. (2010). Thereafter, scats were washed in a sieve with 0.5 mm mesh to clean the undigested, macroscopic scat remainders. The scat remainders were dried on plates and then separated into different food categories. We visually estimated the volume of each food item in scats to the nearest 5% so that results could be used to calculate biomass consumed (see below). Food items that were considered trace (<3% of scat) were excluded from analysis to minimize bias (Kamler et al., 2007). For mammals ≥1 kg, hair samples from scats were identified to species by examining the structures of the cuticle and medulla under a microscope, and comparing those to a reference collection of hairs from known species, which we obtained from captive animals or confiscated remains of dead animals. For small (<1 kg) mammals, generally it was not possible to identify remains to species given the great diversity of small mammals that potentially occur in the area (at least 27 species from 16 genera; Lunde & Son, 2001) and their similarities in hair structure. Therefore, we classified small mammal remains to family as either Muridae or Sciuridae based on the tooth morphology. For Muridae teeth, we further grouped these as either small (<2.0 mm) or large (≥2.0 mm) to represent mouse‐sized murids (about 50 g) and rat‐sized murids (about 200 g body mass), respectively.

Results from scat analysis were quantified in terms of the percent biomass consumed, because this method provides the most accurate estimate of carnivore diets, by using correction factors that account for differential digestibility of food items (Klare et al., 2011). Following the recommendations by Klare et al. (2011), we also included percent volume of food items, and the frequency of occurrence (i.e., percentage of scats containing a particular food item) to make our results comparable to previous studies. To calculate percent biomass consumed, we followed Chakrabarti et al. (2016), who developed a generalized model (biomass consumed per collectable scat/predator weight = 0.033 − 0.025exp^−4.284 (weight of prey/predator weight)^) based on feeding trials of lion (*Panthera leo*), leopard, jungle cat, and domestic cat (*F. silvestris catus*).

For weight of prey, we used 100 g for Sciuridae, 50 g for small Muridae (i.e., mice), and 200 g for large Muridae (i.e., rats), based on approximate weights of species within these groups that likely occupy our study site (Lunde & Son, 2001). We assumed a weight of 350 g for birds, which was the approximate weight of Chinese francolin (*Francolinus pintadeanus*), a common gallinaceous bird that occurs in DDF in our study site. We used 100 g for both small lizards and fresh‐water crabs (Potamidae), based on the average weight of specimens we obtained on our study site. We used 2.25 kg for Burmese hare (*Lepus peguensis*), based on the median of the weight range given by Flux and Angermann (1990). The hairs of civets could not be unambiguously assigned to species, therefore we used a live weight of 5.5 kg, which was based on the median of the weight ranges given by Francis (2019) for the four civet species that occurred on our study site. We used 24 kg for muntjac, which was the median of the weight range given by Francis (2019).

To assess seasonal differences in diet, we divided the dry season into the cool‐dry season (Nov‐Feb) and the hot‐dry season (Mar‐May) to parallel major changes in temperature and precipitation. Our study site became inaccessible during the rainy season (Jun‐Oct) because of high water levels and impassable rivers, so we could not collect scats during this season. The cool‐dry season had the lowest average daily temperatures per month (20.9–22.5°C) and lowest average rainfall per month (3–85 mm), whereas the hot‐dry season had the highest average daily temperatures per month (24.3–25.0°C) with increasing average daily rainfall per month (50–306 mm; climate data were from nearby Sen Monorom, Cambodia, 1982–2012; climate‐data.org; accessed on 10 July 2019). For each dry season period, leopard cat scats were pooled across years to obtain minimum samples (>50 scats/season). An insufficient number of scats was obtained for jungle cats to evaluate seasonal differences, therefore only the total was given. To determine if we obtained the minimum number of scats needed to adequately describe the jungle cat diet, we calculated a prey species accumulation curve using the R package Vegan v. 2.5–6 (Oksanen et al., 2019). We calculated the expected mean prey richness and standard deviation with 10,000 permutations to obtain 95% confidence intervals (Gotelli & Colwell, 2001).

To determine if there was a difference in the frequency of consumed prey items between felid species, and if there was a seasonal difference in diet for leopard cats, we used chi‐square contingency table tests. If a significant difference (*p* <.05) occurred, then we used Fisher's exact tests to determine which individual prey categories significantly differed between species or seasons. Based on the biomass of prey categories consumed, we calculated the degree of dietary overlap between the two felid species using Horn's index of overlap (*R*_0_; Krebs, 1989). Based on the results of a concurrent diet study of jackals in SWS (Kamler et al., 2021), we also calculated the degree of dietary overlap between golden jackals (7–10 kg) and both felids to determine the potential competition for prey species among mesocarnivores in SWS. Finally, we calculated Levin's measure of niche breadth (*B*; Krebs, 1989) for each felid species.

### 2.3 Occupancy modelling

We used camera‐trap records of jungle cats and leopard cats, obtained from seven systematic camera‐trap surveys conducted for leopards in SWS from 2009 to 2019, to examine habitat patterns in occupancy and detection probability, and test for possible effects of interactions in their spatial patterns of occurrence. Camera traps were set typically 2–3 km apart (mean = 2.4 km) along dirt tracks and trails within the core zone of SWS, typically for 3 months during the dry season. Each camera station consisted of paired cameras placed on opposite sides of the trail and attached to trees 2–3 m from the middle of the trail (for more details see Rostro‐García et al., 2018). Camera‐trap grids approximated a systematic array, adjusted to the logistical circumstances, to cover parts of the core zone of SWS, with approximately 64% of the cameras in DDF and 36% in evergreen forests. We adopted the multi‐species occupancy model of Waddle et al. (2010), which given its hierarchical nature, allowed us to simultaneously estimate occupancy and detection parameters for both species, and possible effects of species interactions. We included the effects on occupancy of two habitat covariates: distance to water and habitat type (i.e., DDF or evergreen forests) using GIS layers obtained from WWF‐Cambodia. We further modelled the potential effect of jungle cat (dominant species) presence on leopard cat (subordinate species) occupancy and detection. We relaxed occupancy model assumptions by interpreting occupancy probability as probability of site use (Mackenzie et al., 2018). In addition, we accounted for varying effort due to theft or camera malfunctioning by including the number of days each station was functional within a 15‐day occasion as a covariate on detection for both species. Prior to modelling, we standardized the distance to water to a zero mean and unit standard deviation.

We calculated Bayesian *p*‐values to test if the habitat use model adequately fit the data (Gelman et al., 1996), with values close to 0.5 indicating model fit. We obtained this value by calculating a fit statistic (i.e., residual) that depended on the model parameters and the observed data. We obtained the same fit statistic for a simulated set of data generated from the model under consideration, and then calculated the proportion of times the residuals from the newly generated data were smaller or larger than those from the original data. We used Freeman–Tukey residuals, *R*, such that:

$$R\left(y,\theta \right)=\sum {\left(\sqrt{y}-\sqrt{E\left(y\right)}\right)}^{2}$$

where *y* is the relative detection frequency, θ represents all parameters in the habitat use model, and *E*(*y*) is the expected value of *y*, in this case, the product of site and occasion specific detection and site use probabilities. Occupancy models assumed spatial independence among sampling sites. Given that the studied species have average home range diameters generally smaller than the average camera‐trap spacing of the surveys which targeted leopards, we considered spatial independence not to be an issue. We implemented the model in a Bayesian framework using JAGS (Plummer, 2003) through the R package jagsUI ver. 1.5.1 (Kellner, 2019). We ran three parallel Markov chains with 250,000 iterations each, of which we discarded 50,000 as burn‐in, and we thinned the remaining iterations by 20 to make the output more manageable. For each parameter we assessed chain convergence using the Gelman–Rubin statistic, assuming that values *R̂* < 1.1 indicated convergence (Gelman et al., 2004), and visually inspected the time series plots. We report the results as posterior mean and standard deviation, and 95% (i.e., the 2.5% and 97.5% percentiles of the posterior distribution) Bayesian credible intervals (BCI). We considered a coefficient to have strong support if the 95% BCI did not overlap zero.

### 2.4 Temporal analysis

We examined the diel activity patterns of jungle cats and leopard cats from the camera‐trap data using circular statistics, by constructing models that predicted daily activity as a function of continuous trigonometric predictor variables (Frey et al., 2017). To avoid inflated counts caused by repeated detection of the same event, photos of the same species were considered notionally independent events if they were >30 min apart (O'Brien et al. (2003). The predictor variables described one (sinθ, cosθ) and two (sin2θ, cos2θ) complete cycles in a 24‐hr period, with θ = π *t*/24, where *t* is the time in hours (Ross et al., 2013). To test whether the activity cycles of jungle cats and leopard cats differed, we used species as a categorical predictor and conducted an ANOVA test. Patterns were visualized using the R package plotrix (Lemon, 2006).

We examined jungle cat and leopard cat activity patterns and measured the overlap between these species using the method developed by Ridout and Linkie (2009) implemented in the R package overlap v. 0.3.3 (Meredith & Ridout, 2018). To assess the activity patterns, we used nonparametric circular kernel density functions, where the observed capture times were regarded as random samples from underlying continuous distributions (Ridout & Linkie, 2009). The coefficient of overlapping (Δ), which ranged from 0 (no overlap) to 1 (complete overlap), was determined as the area lying under both species’ density functions. We used the nonparametric estimator of the coefficient of overlapping ${\hat{\Delta }}_{1}$ and calculated a 95% confidence interval of the overlapping index by generating 10,000 smoothed bootstrap samples (Meredith & Ridout, 2018) by first fitting a kernel density to the original data and then drawing random simulated observations from this distribution. To account for the successive changes of the sun's position throughout the year in the celestial sphere (Nouvellet et al., 2012), we determined sunrise and sunset times based on the dates and locations, and for each record mapped sunrise and sunset to π/2 and 3π/2, respectively. Following Lynam et al. (2013), we considered ${\hat{\Delta }}_{1}$ ≥ 0.70 and ${\hat{\Delta }}_{1}$ ≤ 0.35 as high and low overlap of diel activity, respectively. All analyses were performed in R version 3.6.1 (R Core Team, 2019).

## 3 RESULTS

### 3.1 Diets

We collected 196 presumed small felid scats, and DNA analysis confirmed that 130 scats were from leopard cats, 17 from jungle cats, 1 from a jackal, and 48 that failed to determine a species. Mean (±*SD*) scat diameter was significantly larger (*t*_43_ = −2.14, *p* =.038) for jungle cats (2.0 ± 0.2 cm; range = 1.8–2.3 cm) than leopard cats (1.8 ± 0.2 cm; range = 1.5–2.5 cm). The prey accumulation curve for jungle cats reached an asymptote after 9 scats for both the actual and simulated data (Data S1), indicating our sample size was sufficient to describe the jungle cat diet in our study area. The relatively low number of scats needed to reach an asymptote likely was due to the relatively low diversity of prey groups available to jungle cats. The diet of jungle cats was comprised mostly of murids (56%), followed by sciurids (15%), hares (12%), birds (8%), and small reptiles (8%; Table 2). The diet of leopard cats was comprised mostly of murids (73%), followed by sciurids (17%; Table 2). Overall diets differed between the species (*X* ^2^ = 17.15, *p* =.009) because jungle cats consumed large murids (*p* =.048), birds (*p* =.046), hares (*p* =.013), and reptiles (*p* <.001) more frequently than leopard cats. Niche breadth (*B*) was broader for jungle cats (5.09) than leopard cats (3.31; Table 2). Despite this, the dietary overlap between these species was relatively high (*R*_0_ = 0.85) primarily because of the high consumption of small rodents (murids and sciurids) by jungle cats (71% biomass consumed) and leopard cats (90%; Table 2). In contrast, the dietary overlap was relatively low between jungle cats and jackals (0.31), and between leopard cats and jackals (0.32).

**TABLE 2:** Diet composition expressed as percentage of ingested biomass (Bio), percentage of scat volume (Vol), and frequency of occurrence (Occ) of jungle cats (*Felis chaus*) and leopard cats (*Prionailurus bengalensis*) in Srepok Wildlife Sanctuary, Cambodia, 2013–2016 (*n* = number of scats analyzed). Seasonal and total results are given for leopard cats. Dietary niche breadth (*B*) is given based on biomass consumed

Prey category	Jungle cat			Leopard cat								
	Total (*n* = 17)			Cool‐dry (*n* = 57)			Hot‐dry (*n* = 73)			Total (*n* = 130)		
	Bio	Vol	Occ	Bio	Vol	Occ	Bio	Vol	Occ	Bio	Vol	Occ
Small rodent	71.3	78.8	100.0	91.4	95.2	98.2	89.3	93.0	98.6	90.2	94.0	98.5
Muridae	56.0	62.1	88.2	69.2	72.9	78.9	75.9	79.7	87.7	72.9	76.7	83.8
Small species	33.7	40.9	64.7	44.2	50.4	63.2	52.0	58.2	64.4	48.6	54.8	63.9
Large species	17.6	16.2	29.4	10.6	8.8	15.8	6.7	5.5	8.2	8.5	6.9	11.5
Unknown size	4.8	5.0	5.9	14.4	13.7	14.0	17.2	16.1	17.8	15.9	15.0	16.2
Sciuridae	15.3	16.7	47.1	22.2	22.3	28.1	13.4	13.3	20.5	17.3	17.2	23.8
Burmese hare (*Lepus peguensis*)	11.5	4.3	11.8	0.0	0.0	0.0	0.0	0.0	0.0	0.0	0.0	0.0
Muntjac (*Muntiacus vaginalis*)	0.0	0.0	0.0	0.0	0.0	0.0	4.1	1.3	1.4	2.3	0.7	0.8
Civet^a^	0.0	0.0	0.0	5.2	1.7	1.8	0.0	0.0	0.0	2.3	0.7	0.8
Bird	8.4	6.5	23.5	1.4	1.0	5.3	4.4	2.9	8.2	3.1	2.1	6.9
Small reptile	8.0	8.7	64.7	2.0	2.0	22.8	2.1	2.1	15.1	2.1	2.1	18.5
Fresh‐water crab (Potamidae)	0.8	0.9	11.8	0.0	0.0	0.0	0.0	0.0	0.0	0.0	0.0	0.0
Insect	—	0.9	17.6	—	0.2	1.8	—	0.6	12.3	—	0.4	8.5
Niche breadth (*B*)	5.09			3.57			3.06			3.31		

^a^ Asian palm civet (*Paradoxurus hermaphroditus*), small Indian civet (*Viverricula indica*), large Indian civet (*Viverra zibetha*), or large‐spotted civet (*Viverra megaspila*).

### 3.2 Occupancy models

We obtained 341 independent records of both species from 450 camera‐trap stations totaling 29,837 trap nights from 2009 to 2019. Leopard cats had the highest number of independent records (*n* = 288), accounting for 84.5% of all small felid detections, compared to 15.5% (*n* = 53) of jungle cats. Leopard cats were detected at about four times more stations (*n* = 139; 30.9% of stations) than jungle cats (*n* = 32; 7.1% of stations).

The probability of habitat use (i.e., occupancy) by jungle cats was strongly and negatively associated with evergreen forests, suggesting higher habitat use of DDF by this species. In contrast, the effects of habitat type on habitat use of leopard cats were weak, with BCI encompassing zero, indicating no habitat preference for the species. Habitat use of jungle cats and leopard cats decreased as cameras were set farther away from water sources, although the effects for both species were weak with both BCIs encompassing zero. Jungle cats were estimated to use 12.2% of the camera‐trap sites (5% greater use than the observed proportion of habitat use), whereas leopard cats were estimated to use 56.5% of the camera‐trap sites (25.6% greater than the observed proportion of habitat use). We found slight evidence of spatial avoidance of jungle cats by leopard cats, indicated by the negative log‐odd value, although the BCI largely encompassed zero. Contrary to this, leopard cats were more likely to be detected at stations where jungle cats were detected, with more than 90% of the probability mass being positive. Overall, the detection rate increased with effort. The Gelman–Rubin diagnostic indicated adequate convergence of all parameters, and the Bayesian *p*‐values of the model adequately described the data.

### 3.3 Temporal patterns

**FIGURE 2:**
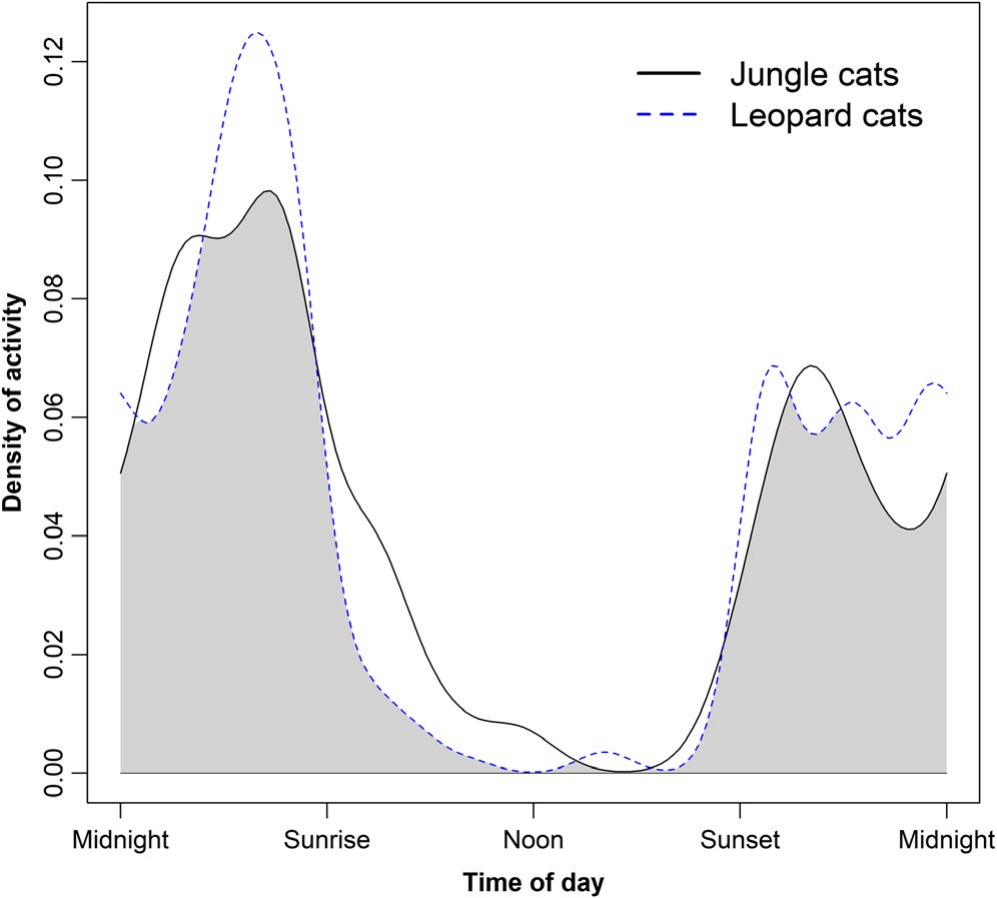
Kernel density estimates of diel activity patterns of jungle cats (*Felis chaus*) and leopard cats (*Prionailurus bengalensis*) based on the time of independent camera‐trap photographs obtained from 2009 to 2019 in Srepok Wildlife Sanctuary, Cambodia. The coefficient of overlapping (0.86, 95% CI: 0.77–0.93) is represented by the gray shaded area

The results from the wave analysis suggested that there was no significant difference in activity between the species (*p* > .05). Both jungle cats and leopard cats were mostly nocturnal, with only 15.1% and 5.6% of records during daylight, respectively. Accordingly, diel activity of leopard cats strongly overlapped that of jungle cats (${\hat{\Delta }}_{1}$ = 0.86, 95% CI = 0.77–0.93; Figure 2), indicating a high temporal overlap between the species.

## 4 DISCUSSION

We conducted the first dietary study of jungle cats in Southeast Asia, within one of the largest tracts of threatened DDF remaining in the region. In addition, our study provided information about jungle cat ecology and its interaction with a sympatric felid, adding to the pool of knowledge of the species within this globally important habitat type. Overall, there was high niche overlap between jungle cats and leopard cats in DDF because the two species did not exhibit strong dietary, temporal, or spatial partitioning. Although we could not determine if jungle cats and leopard cats were competing for limited food resources, dietary overlap between the species was relatively high in the dry season, which did not support our prediction. The primary reason for the high dietary overlap was the high consumption of small rodents by both jungle cats and leopard cats. However, the overall diets differed between species because jungle cats consumed hares, large murids, birds, and reptiles more frequently than leopard cats. Also contrary to our prediction, remains of small ungulates were not found in any jungle cat scats, indicating jungle cats did not predate on ungulate fawns, at least during the dry season. Concurrent dietary studies in SWS showed that ungulates comprised 41% of the jackal diet (Kamler et al., 2021), 87% of the leopard diet (Rostro‐García et al., 2018), and 92% of the dhole diet (Kamler, Thatdokkham, et al., 2020), indicating jungle cats also did not scavenge from kills of larger carnivores during the dry season. Although consumption of small ungulates cannot be ruled out completely given our small sample size of scats from only the dry season, our results were in contrast to several studies in India, which showed that jungle cats regularly consumed ungulate fawns or ungulate carrion (Table 1). Instead, jungle cats in SWS consumed a diverse array of small prey, including rodents, birds, and reptiles, similar to that reported in previous dietary studies from southwestern Asia (Table 1). With the exception of hares, all prey species of jungle cats were <1 kg, which was consistent with previous conclusions that jungle cats typically prey on species <1 kg (Sunquist & Sunquist, 2002). In fact, the jungle cat is likely a small rodent specialist because, like the serval (*Leptailurus serval*), it has long legs, a slender build, a small head, and tawny pelage which are considered adaptations for preying on small rodents in grassland habitat (Nowell & Jackson, 1996).

Our study of jungle cats was the first to use DNA analysis to confirm scats to this species, and results showed that the range of scat diameters overlapped for both jungle cats and leopard cats. Additionally, scat diameters of both felid species overlapped those of DNA‐confirmed scats from jackals (1.8–3.2 cm) based on a concurrent study in SWS (Kamler et al., 2021). These results indicate that scat size alone cannot be used to distinguish among mesocarnivores in Asia. Considering how common it is for field researchers to misidentify carnivore scats (Baines et al., 2013; Janečka et al., 2008; Karmacharya et al., 2016; Khatoon et al., 2019; Weiskopf et al., 2016), we recommend that genetic analyses be used in all future studies that investigate the diets of small felids and other sympatric carnivores.

Our results were consistent with previous studies that showed leopard cats are murid specialists, and only consume prey <500 g (see Kamler, Inthapanya, et al., 2020 for a review of leopard cat diets). The only exceptions in our study were two scats that contained a fraction of muntjac and civet remains, respectively, suggesting they represented rare scavenging events (Klare et al., 2014). Leopard cats consumed a higher biomass of murids than jungle cats, but consumed less large murids than did jungle cats, indicating leopard cats preyed on smaller species than did jungle cats. In general, both felid species appeared to be specialists on small rodents, although jungle cats had a higher niche breadth and consumed slightly larger prey species than leopard cats, probably because the larger body size of jungle cats allowed it to take a wider variety of prey sizes (Gittleman, 1985). Both felids had low dietary overlap with jackals, a species that consumed mostly ungulates, termites (*Hospitalitermes* spp.), and civets in SWS during the dry season (Kamler et al., 2021). This indicates that jungle cats and leopard cats did not compete with jackals, a larger mesocarnivore (7–10 kg; Kamler et al., 2021), for the same food resources, which likely helped facilitate their coexistence with this potentially dominant competitor. Due to the field conditions, we were unable to collect scats during the rainy season, which likely was a time of abundant food resources for both small felid species. Therefore, dietary overlap and food habits may have been different during the rainy season, although further research is required to test this hypothesis.

The activity patterns had high overlap and did not differ between jungle cats and leopard cats, which did not support our prediction that they should have low activity overlap. Our results showed that leopard cats were almost strictly nocturnal, which is consistent with several previous studies (Grassman et al., 2005; Kamler, Inthapanya, et al., 2020; Lynam et al., 2013; McCarthy et al., 2015; Mukherjee et al., 2019), indicating the activity pattern of this felid is relatively consistent across its distribution. However, our results also showed that jungle cats were almost strictly nocturnal, which was unexpected because previous studies reported that jungle cats were more diurnal than other felids (Gray et al., 2014; Lekagul & McNeely, 1977; Nowell & Jackson, 1996; Sunquist & Sunquist, 2002). Their nocturnal activity was not likely due to avoidance of larger carnivores because leopards, dholes and jackals also were nocturnal in SWS (Kamler et al., 2021; Rostro‐García, Panthera, WildCRU, WWF‐Cambodia, unpubl. data). The camera‐trapping surveys were carried out during the dry season when monthly temperatures are the highest and the grassy understory burns, which may have caused the jungle cat to favor nocturnal activity during this season. It is possible that one or both felid species could have become more diurnal during the rainy season, given the greater herbaceous cover and lower daily temperatures during this period. Alternatively, illegal human activity, which is primarily diurnal, has been increasing in SWS (Rostro‐García et al., 2018); thus, jungle cats may have become nocturnal to avoid humans, similar to that observed in other species (Gaynor et al., 2018). Future research is needed to investigate whether seasonal and human‐induced changes in activity patterns occur for jungle cats in DDF.

The occupancy analysis showed that jungle cats used DDF almost exclusively, which supported our prediction and confirmed that this species is a DDF specialist in Southeast Asia. The avoidance of evergreen habitat by jungle cats was consistent with previous camera‐trap surveys conducted in Cambodia (Gray et al., 2014; Suzuki et al., 2017), and suggests that the specialization of jungle cat for hunting in open habitats precludes it from regularly using closed evergreen forests. This is even more remarkable given that evergreen forests in Southeast Asia contain a higher biomass of small rodents compared to nearby DDF (Petersen et al., 2019; Walker & Rabinowitz, 1992). Additionally, because the understory of the evergreen forests does not burn during the dry season to the same extent as DDF, small rodent numbers in evergreen forests are more stable and remain about five times higher than those in nearby DDF after dry season fires (Walker & Rabinowitz, 1992). Despite the potentially larger number of small rodents in evergreen forests, especially after the dry season fires, jungle cats continued to use DDF almost exclusively, suggesting that prey abundance alone does not affect the habitat use of jungle cats.

Habitat use of leopard cats in SWS was not affected by forest type, which did not support our prediction. Although leopard cats are habitat generalist (Ross et al., 2015), we had predicted that this species would use mostly evergreen forests in SWS to avoid competition with jungle cats, which are DDF specialists. Additionally, we assumed that leopard cats would be more attracted to evergreen forests compared to DDF because of the higher numbers and biomass of small rodents in evergreen forests. Instead, leopard cat occupancy was not affected by habitat type in SWS, regardless of potential differences in prey biomass and competitors. Our results provide further confirmation that leopard cats are a behaviorally flexible habitat generalist (Ross et al., 2015), and appear to use habitats in proportion to availability. Habitat use differences (i.e., specialized versus generalized) might have been the primary mechanism of coexistence between jungle cats and leopard cats on our study site.

Overall, the estimated proportion of camera stations used by leopard cats was more than 4 times higher than jungle cats, which did not support our prediction. We had predicted a higher occupancy of the jungle cat, a DDF specialist, because DDF dominated our study site. The estimated occupancy and total number of detections for each species were similar to the percent of DNA‐confirmed scats we collected on the study site, as 88% of small felid scats were from leopard cats compared to only 12% from jungle cats. Therefore, the data from both scats and camera traps indicated that leopard cats likely were more abundant than jungle cats in SWS, possibly due to differences in habitat use between the species. The use of evergreen habitat by leopard cats likely resulted in a higher carrying capacity of this species on our study site, given the larger number of rodents and lack of fires in this habitat compared to DDF. Our results are consistent with a concurrent study of jackals in SWS, which showed this species also is a DDF specialist that avoided evergreen forests and occurred at extremely low density (Kamler et al., 2021). The harsh environmental conditions, fires, and dramatic decrease in small vertebrate prey in DDF during the dry season likely results in a low carrying capacity for several mesocarnivore species that are DDF specialists.

The occupancy analysis suggested that leopard cats seemed to avoid jungle cats but were more easily detected in areas where jungle cats were detected. This seemingly contradictory result likely was caused by a higher abundance of leopard cats, and their nonpreference for any habitat type. Overall, these results did not support our prediction that leopard cats would spatially avoid jungle cats. The apparent low abundance of jungle cats on our study site may have prevented this species from having a strong population‐level effect on leopard cats. Furthermore, leopard cats may have avoided jungle cats at finer scales not investigated in this study, such as the level of the home range, feeding site, or resting site (Broekhuis et al., 2013; Rostro‐García, Tharchen, et al., 2016; Rostro‐García et al., 2015). Alternatively, interspecific interactions, which vary among carnivore species (Donadio & Buskirk, 2006), are not always related to negative associations, even between species that are similar in body size and taxonomy (Boron et al., 2020; Davis et al., 2011; Gutiérrez‐González & López‐González, 2017; Kamler et al., 2012; Loveridge & Macdonald, 2002). Thus, perhaps jungle cats were not physically aggressive toward leopard cats and did not behaviorally dominate them, although further research is required to test this hypothesis. Additionally, future research should consider multi‐scale and seasonal differences in the ecology of these species, because their interactions might change across different scales and seasons.

### 4.1 Implications for conservation of jungle cats in Southeast Asia

There are three recognized subspecies of jungle cats, including *F. chaus fulvidina* which only occurs in Southeast Asia (Kitchener et al., 2017). This is the only jungle cat subspecies that is a conservation concern because it has become one the rarest felids in Southeast Asia and only survives in small isolated populations within suitable habitat (Duckworth et al., 2005). Jungle cats might be able to occur in open scrub and other secondary habitats in Southeast Asia, but due to anthropogenic pressures these habitats are now unusable by them (Duckworth et al., 2005), leaving DDF as the only major remaining suitable habitat for jungle cats. Our study, carried out in the last stronghold of jungle cats in the region, and in one of largest remaining tracts of DDF, suggests this species might be rare not only because of poaching and limited availability of DDF, but also likely because of the seemingly low carrying capacity of jungle cats in this habitat owing to its harsh dry season conditions. Consequently, our results indicate that relatively large areas of DDF will be required for the long‐term conservation of this rare felid in Southeast Asia. Unfortunately, DDF is easily accessible to humans, who are increasingly causing habitat destruction and overhunting species within it (Duckworth et al., 2005; Rostro‐García et al., 2018). Thus, immediate conservation action should be taken to conserve jungle cats in Southeast Asia, primarily by making and implementing conservation plans which should include the protection of large tracts of DDF and increased law enforcement activities for these areas. Only with implementation of better conservation strategies for DDF will the long‐term conservation of jungle cats and other DDF specialists be feasible in Southeast Asia.

## CONFLICT OF INTEREST

The authors declare that they have no competing interests.

## AUTHOR CONTRIBUTIONS

**Susana Rostro‐García:** Conceptualization (lead); Data curation (lead); Formal analysis (lead); Investigation (lead); Methodology (lead); Project administration (lead); Writing‐original draft (lead); Writing‐review & editing (lead). **Jan F Kamler:** Methodology (equal); Writing‐original draft (supporting). **Christin Minge:** Methodology (supporting). **Anthony Caragiulo:** Methodology (supporting); Writing‐review & editing (supporting). **Rachel Crouthers:** Methodology (supporting); Writing‐review & editing (supporting). **Milou Groenenberg:** Methodology (supporting); Writing‐review & editing (supporting). **Thomas Gray:** Methodology (supporting); Writing‐review & editing (supporting). **Visattha In:** Methodology (supporting). **Chanratana Pin:** Methodology (supporting); Writing‐review & editing (supporting). **Prum Sovanna:** Methodology (supporting). **Marc Kéry:** Formal analysis (supporting); Writing‐review & editing (supporting). **David Macdonald:** Funding acquisition (equal); Supervision (supporting); Writing‐review & editing (supporting).

## Supporting information

Data S1

## Data Availability

Because of conservation reasons, the authors are unable to share the camera‐trap locations as the data derives from endangered species surveys. For access to additional data presented in this study, please contact Susana Rostro‐García from the Wildlife Conservation Research Unit, University of Oxford (rostro.susana@gmail.com).
