# Supplementary material for: Small cats in big trouble? Diet, activity, and habitat use of jungle cats and leopard cats in threatened dry deciduous forests, Cambodia
Source: Ecol Evol. 2021 Mar 30;11(9):4205–17. doi: 10.1002/ece3.7316 (PMC8093725; doi:10.1002/ece3.7316)

Supplementary Data S1 – Accumulation curve (grey line) of expected mean prey richness from jungle cat scats collected in Srepok Wildlife Sanctuary, Cambodia, 2013-2016. The bars represent 95% confidence intervals based on 10,000 permutations. The black line represents the actual accumulation of prey species based on scat content in the order in which scats were found.

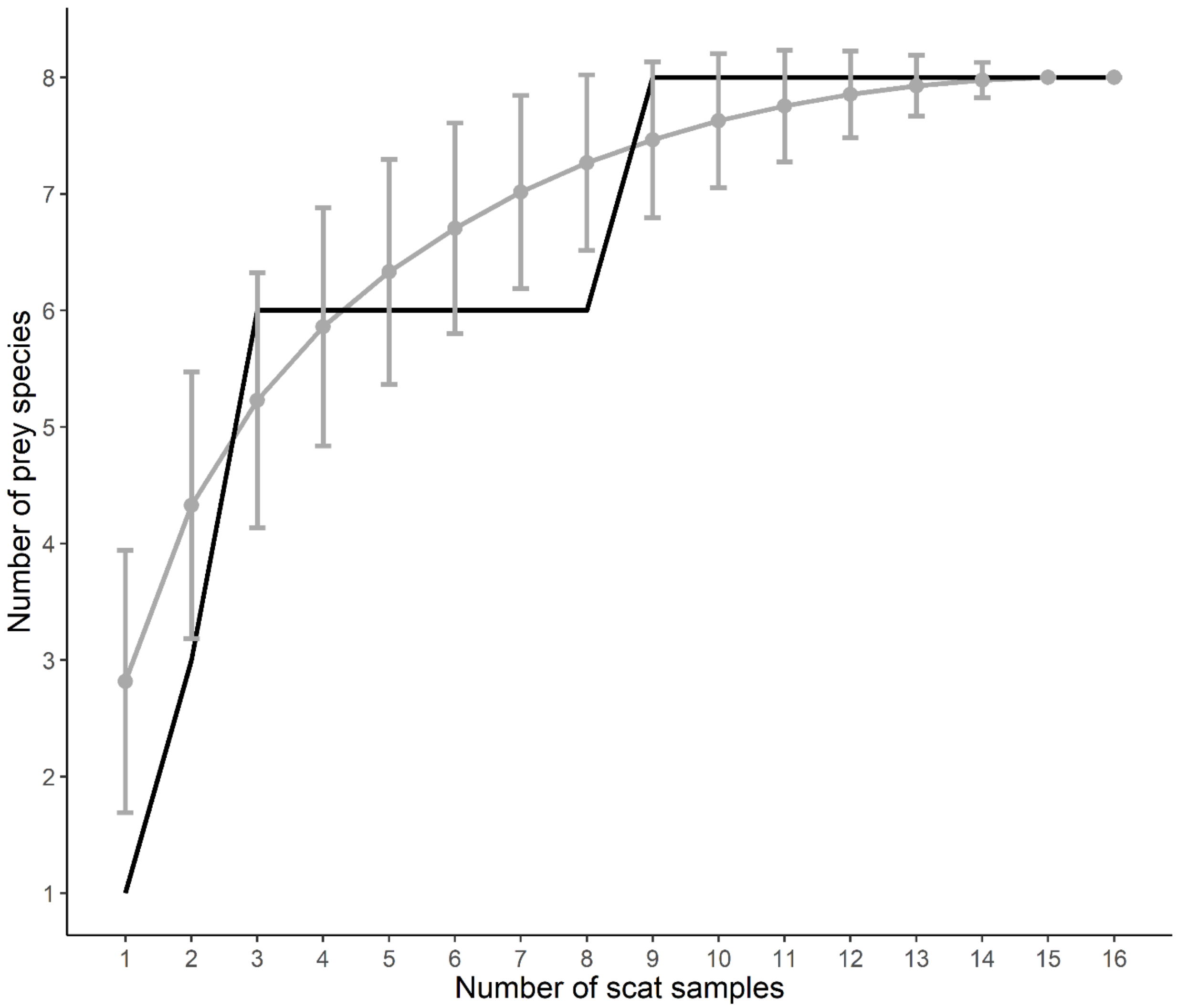

Supplement: Supplementary file 1 — Data S1 [file ECE3-11-4205-s001.pdf]
